# Punicalagin Regulates Key Processes Associated with Atherosclerosis in THP-1 Cellular Model

**DOI:** 10.3390/ph13110372

**Published:** 2020-11-08

**Authors:** Sanaa Almowallad, Etimad Huwait, Rehab Al-Massabi, Salma Saddeek, Kalamegam Gauthaman, Alexandre Prola

**Affiliations:** 1 Department of Biochemistry, Faculty of Sciences, King Abdul Aziz University, Jeddah 21589, Saudi Arabia; rf-saif@ut.edu.sa (R.A.-M.); salmayms@uhb.edu.sa (S.S.); 2 Cell Culture Unit, King Fahad Medical Research Centre, King Abdul Aziz University, Jeddah 22252, Saudi Arabia; 3 Department of Biochemistry, Faculty of Sciences, University of Tabuk, Tabuk 71491, Saudi Arabia; 4 Department of Chemistry, Faculty of Sciences, University of Hafr Al Batin, Hafr Al Batin 31991, Saudi Arabia; 5 Center of Excellence in Genomic Medicine Research, King Abdulaziz University, Jeddah 21589, Saudi Arabia; Kgauthaman@kau.edu.sa; 6 Department of Medical Laboratory Technology, Faculty of Applied Medical Sciences, King Abdulaziz University, Jeddah 21589, Saudi Arabia; 7 Department of Cell Physiology and Metabolism, Faculty of Medicine, University of Geneva, 1 rue Michel-Servet, CH-1211, 1202 Geneva, Switzerland; alexandre.prola@unige.ch

**Keywords:** atherosclerosis, punicalagin, inflammation, monocyte migration, cholesterol efflux

## Abstract

Atherosclerosis may lead to cardiovascular diseases (CVD), which are the primary cause of death globally. In addition to conventional therapeutics for CVD, use of nutraceuticals that prevents cholesterol deposition, reduce existing plaques and hence anti-atherosclerotic effects of nutraceuticals appeared to be promising. As such, in the present study we evaluated the beneficial effects of punicalagin, a phytochemical against an atherosclerotic cell model in vitro. Cytotoxicity assays were examined for 10 µM concentration of punicalagin on THP-1 macrophages. Real-time-polymerase chain reaction (RT-PCR) was used to analyze monocyte chemoattractant protein-1 (MCP-1) and Intercellular adhesion molecule (ICAM-1) expressions. Monocyte migration and cholesterol efflux assays were performed to investigate punicalagin’s further impact on the key steps of atherosclerosis. Cytotoxicity assays demonstrated no significant toxicity for punicalagin (10 µM) on THP-1 macrophages. Punicalagin inhibited the IFN-γ-induced overexpression of MCP-1 and ICAM-1 in macrophages by 10 fold and 3.49 fold, respectively, compared to the control. Punicalagin also reduced the MCP-1- mediated migration of monocytes by 28% compared to the control. Percentages of cellular cholesterol efflux were enhanced in presence or absence of IFN-γ by 88% and 84% compared to control with 58% and 62%, respectively. Punicalagin possesses anti-inflammatory and anti-atherosclerotic effects. Punicalagin also did not exhibit any cytotoxicity and therefore can be considered a safe and potential candidate for the treatment and prevention of atherosclerosis.

## 1. Introduction

Currently, cardiovascular-related diseases (CVD) remain the leading cause of death globally in comparison to other chronic illnesses. In 2012, CVD was responsible for 31%, or 17.5 million deaths across the globe, and the number of fatalities is estimated to rise to approximately 23.3 million within the next decade [1]. Moreover, the increased prevalence of CVD poses considerable burdens for healthcare systems due to the high costs involved in treating and managing the condition [1,2]. Atherosclerosis remains the primary cause of deadly heart diseases [3] and occurs when long-lasting inflammation affects the vascular system.

Atherosclerosis is a progressive illness that results from the accumulation of fatty acids and lipids in medium- and large-sized arteries, which then become precursors to the buildup of plaque [4]. Over time, size of plaque increase and may eventually rupture, releasing vast amounts of necrotic fragments into the bloodstream, thus increasing the probability of blood clots leading to myocardial infarctions, stroke, or both [5].

Atherosclerosis treatment includes the administration of statin as a main core element. However, a significant issue with its use concerns increased associations with a continued risk of CVD, as well as causing severe, adverse side effects such as diabetes [6] or hemorrhagic stroke [7] in different patient populations. Current studies examining other pharmaceutical agents for atherosclerosis treatment have, thus far, yielded disappointing results during clinical trials. In this context, the development of nutraceuticals appears to be promising to prevent the development of atherosclerosis. The THP-1 macrophage cell line is thought to be an interesting model for studying the anti-atherosclerotic potential of many natural products [8,9].

Punicalagin is a phytochemical polyphenolic compound extracted from pomegranate (*Punica granatum*) and is the most abundant polyphenol in pomegranate juice (80% *w*/*w*) [10]. Punicalagin is known to have strong antioxidant characteristics in comparison to other nutraceuticals [3,11]. Studies on patients undergoing hemodialysis demonstrated that pomegranate juice did not affect or alter inflammatory markers expression after six months of about 1000 mg per day uptake [12]. Another study has reported that cholesterol level and oxidized low-density lipoprotein (LDL) were found to be reduced after consuming pomegranate juice [13]. However, past research has prioritized the effects of pomegranate crude extracts [14] or juice [13] and has not investigated the anti-atherogenic effects of punicalagin alone. It is well-known that pomegranate is a great source of antioxidants and therefore were used for treating heart disease. However, pomegranate has been used traditionally in treating many health conditions such as pancreatic inflammation, vaginal discharge, diabetes, and diarrhea [15]. As a result, the physiological activity of punicalagin on various vital atherosclerosis episodes remains currently poorly understood.

Monocyte chemoattractant protein-1 (MCP-1) is a chemokine known for its ability to recruit monocytes and macrophages to the site of inflammation [16]. Intercellular adhesion molecule-1 (ICAM-1) is involved in contacts with other cells or with the extracellular matrix [17] and enables macrophages to migrate towards inflammation sites [18]. Previous studies suggest that MCP-1 and ICAM-1 are essential markers in several vascular diseases, among which is atherosclerosis, by recruiting monocytes and favoring the adhesion of macrophages to the active endothelium [5,18,19].

The aim of this study was to investigate markers for atherosclerosis, including inflammation and lipid deposition, in response to punicalagin in classically activated type 1 macrophages (M1) differentiated from THP-1 monocytic cell line. In addition, we investigated human monocyte migration and cholesterol efflux to assess the effect of punicalagin on atherosclerosis progression.

## 2. Results

### 2.1. Punicalagin Does Not Alter THP-1 Macrophages Viability

**Figure 1:**
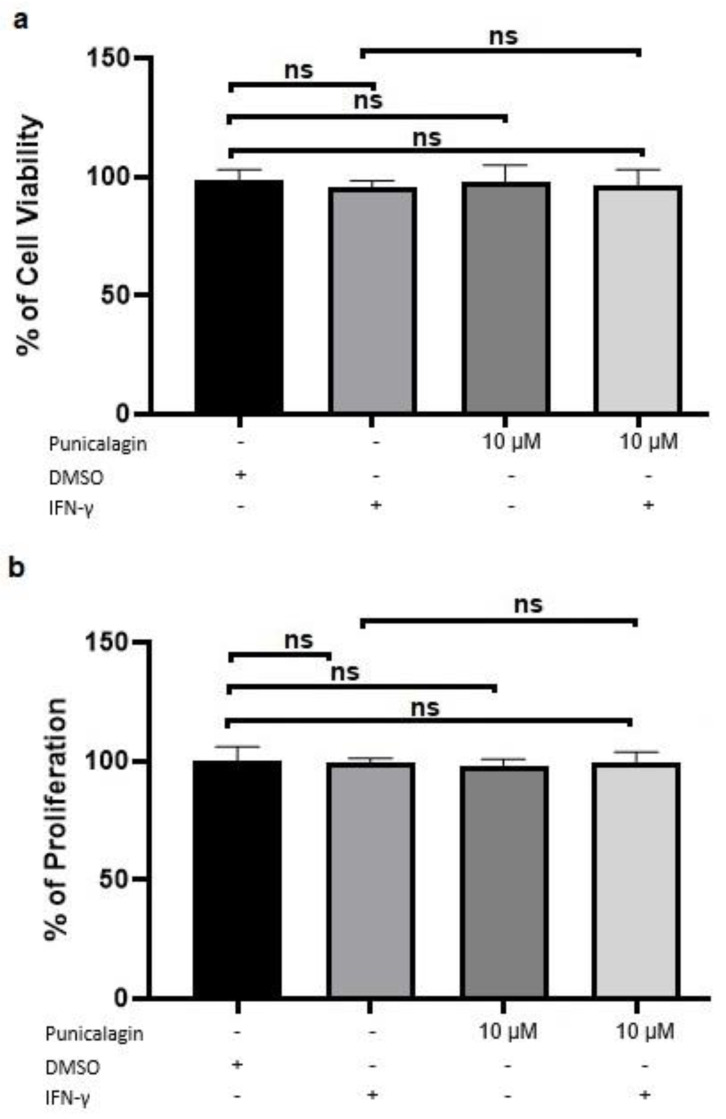
Analysis of punicalagin-induced cytotoxicity on THP-1 macrophages. Treatment of cells with 10 μM of punicalagin or dimethyl sulfoxide (DMSO) was conducted for 24 h. Values are represented as mean of three independent experiments +/− SEM. (**a**) Lactate dehydrogenase release was measured by removing media from the treated plates. (**b**) Remaining macrophages were used for the crystal violet assay. Values of average percentage of dead cells and cell viability are displayed in comparison to vehicle, which was set at 1. ns = non-significant.

Punicalagin-induced cytotoxicity was measured by lactate dehydrogenase (LDH) assays whereas cell viability was measured by crystal violet using 10 μM concentration of punicalagin on THP-1 macrophages. LDH is a cytoplasmic enzyme released in the extracellular medium when plasma membrane is damaged. Therefore, LDH release is commonly used to assess cytotoxicity. As shown in Figure 1a, there was no significant release of LDH by the macrophages following incubation with punicalagin compared to vehicle-treated cells. Crystal violet binds to the DNA of adherent cells; thus, the staining correlates with cell number. Detached cells were considered as non-viable and discarded before quantification. Punicalagin treatment induced no significant difference in macrophage viability compared with vehicle-treated cells (Figure 1b). Furthermore, combined findings from crystal violet and LDH assays, 10 μM concentration of punicalagin was used for further PCR.

### 2.2. Punicalagin Reduces IFN-γ-Induced mRNA Overexpression of MCP-1 and ICAM-1 in THP-1 Macrophages

**Figure 2:**
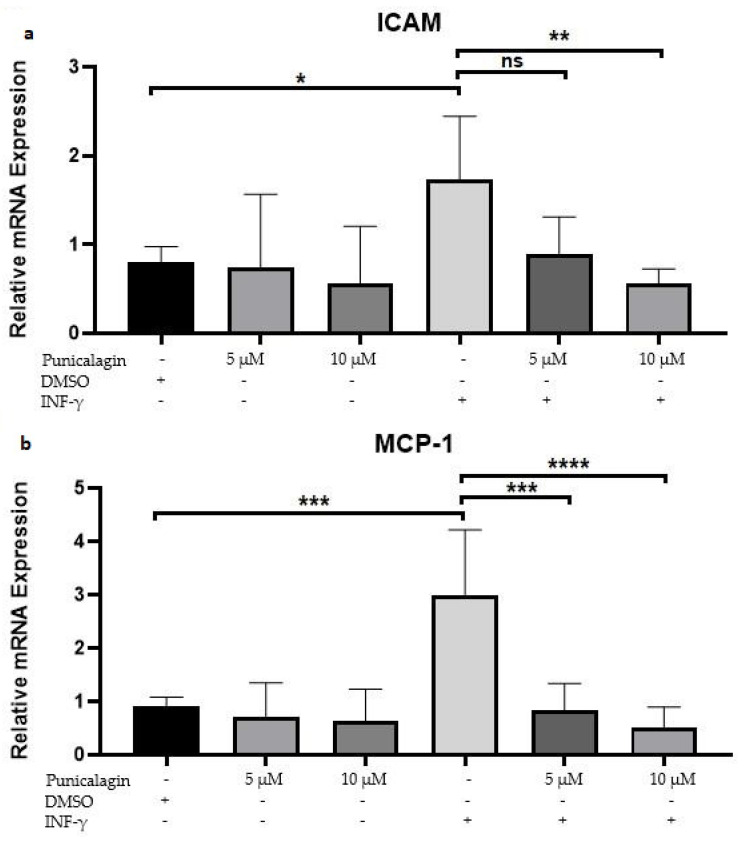
Effect of punicalagin on IFN-γ-induced MCP-1 and ICAM-1 mRNA levels. Macrophages were incubated for 3 h with DMSO or IFN-γ. Cells were treated for 24 h with punicalagin or DMSO alone. RT-qPCR were performed with specific primers to amplify MCP-1 (**a**), ICAM-1 (**b**) and GAPDH complementary DNA (cDNA). The graphs identify the relative fold-changes compared to control, which was assigned to 1. Values are represented as mean of three independent experiments +/− SEM. *: *p* < 0.05, **: *p* < 0.02, ***: *p* < 0.01, ****: *p* < 0.001. ns = non-significant.

THP-1 macrophages were incubated for 3 h with IFN-γ to induce the expression of MCP-1 and ICAM-1 [20]. Subsequently, cells were treated for another 24 h using 5 and 10 μM of punicalagin or with 0.1% of dimethyl sulfoxide (DMSO) as control. IFN-γ induced an overexpression of MCP-1 by 10 and of ICAM-1 by 3.49 (Figure 2). Treatment using 5 and 10 μM of punicalagin resulted in a 38% and 88% reduction of IFN-γ-induced MCP-1 overexpression and in a 79% and 90% reduction of IFN-γ-induced ICAM-1 overexpression, respectively (Figure 2). 

### 2.3. Punicalagin Inhibits MCP-1-Mediated Migration of THP-1 Monocytes

**Figure 3:**
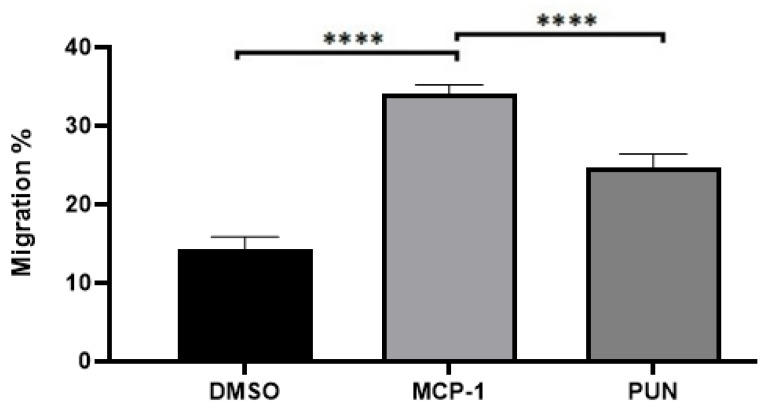
Effect of punicalagin on MCP-1-induced migration of THP-1 monocytes. THP-1 monocytes were treated with DMSO as a vehicle control or with 20 ng/mL MCP-1 or 20 ng/mL MCP-1 with 10 μM of punicalagin for 3 h. Data were normalized to the percentage of cells that migrated from the apical compartment of a modified Boyden chamber into the basolateral compartment in response to MCP-1 alone. Data are presented as mean ± SEM following three independent experiments. Statistical analysis was performed using a one-way ANOVA with a Sidak’s test analysis where ****: *p* < 0.001. PUN = punicalagin.

During the progression of atherosclerosis, the recruitment of monocytes to the affected area is a crucial mechanism underlying lesion formation. Therefore, we analyzed punicalagin’s effect on MCP-1-induced monocytes recruitment. The results of our Boyden chamber experiment, presented in Figure 3, showed a significant increase of 75% in monocyte migration after MCP-1 addition, compared to control sample (*p* < 0.0001). Interestingly, the presence of punicalagin reduced MCP-1-induced monocyte recruitment by 28% (*p* < 0.0001).

### 2.4. Punicalagin Enhances Cholesterol Efflux in IFN-γ-Induced THP-1 Macrophages

**Figure 4:**
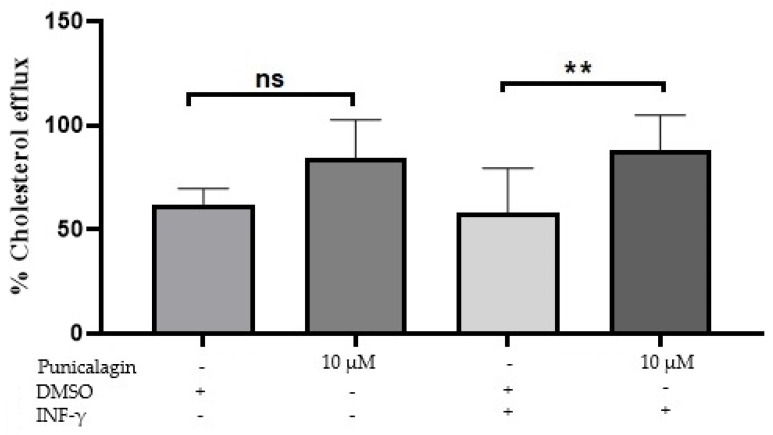
Effect of punicalagin on cholesterol efflux in IFN-γ-induced THP-1 macrophages. THP-1 differentiated macrophages were incubated with or without of IFN-γ for 3 h. THP-1 cells were labeled with florescent cholesterol and incubated overnight. Treatment with 10 μM of punicalagin were added for 24 h. The percentages of cholesterol efflux were then calculated. Data are presented as mean ± SEM following three independent experiments. Statistical analysis was performed using a one-way ANOVA with a Sidak’s test analysis where **: *p* < 0.02, ns = non-significant.

Another key step in atherosclerosis management is cellular cholesterol efflux. Punicalagin seems to have a significant enhancement on cellular cholesterol efflux in THP-1 foam cell model. Results from cholesterol efflux assay presented in Figure 4 showed a non-significant increase in cholesterol efflux in cells treated with 10 μM/mL of punicalagin. However, a significant increase in cholesterol efflux was reported for IFN-γ-induced THP-1 foam cells treated with 10 μM of punicalagin compared to vehicle control. The percentages of cellular cholesterol efflux reported for treatment with 10 μM of punicalagin in the presence or absence of IFN-γ were 54.2% and 51.8%, respectively, with comparison to the percentages of cellular cholesterol efflux of vehicle control in presence or absence of IFN-γ, which are 40% and 36.6%, respectively.

## 3. Discussion

Although the beneficial effect of pomegranates on cardiovascular disease are well-known, the underlying mechanism is poorly understood. The aim of this study is to examine Punicalagin’s anti-atherosclerotic effects on macrophages in vitro. Two key factors leading to atherosclerosis are inflammation [21] or lipid deposition [22]. Based on recent in vivo research, pomegranate has anti-atherosclerotic and anti-inflammatory properties [23]. Our goal in this study is to find out the favored mechanism of action for punicalagin either anti-inflammatory or anti-hyperlipidemic mechanism. We examined the role of punicalagin on pro-inflammatory gene expression and monocyte migration in order to understand punicalagin mechanism of action. Additionally, punicalagin effect on cholesterol efflux process were examined to find out if anti-hyperlipidemic mechanism of action were favored.

Anti-inflammatory effects of pomegranate and/or punicalagin have been widely demonstrated in the literature [24,25,26,27,28]. To study anti-inflammatory effects of punicalagin on THP-1 macrophage, IFN-γ induction were used to accelerate several pro-inflammatory gene expressions, in particular ICAM-1 and MCP-1 [20,24], which contribute to the adhesion and conscription of cells in vitro. During this process, IFN-γ increases the binding of ICAM-1 to endothelial cells [29,30]. In this context, our results interestingly demonstrated the ability of punicalagin to significantly reduce IFN-γ-induced expression of ICAM-1 and MCP-1. This result corroborates a previous study that evidenced MCP-1 down-regulation induced by punicalagin in human intestinal cells [11].

This inhibition of IFN-γ-induced inflammatory signaling by punicalagin is an essential finding, as a default in IFN-γ signaling is associated to the diminution of atherosclerosis lesions in vivo [31]. Thus, our results strongly suggest that IFN-γ signaling is a central target of the anti-atherogenic and anti-inflammatory actions of punicalagin.

Nevertheless, a question remains regarding the molecular pathway through which punicalagin inhibits IFN-γ-induced expression of MCP-1 and ICAM-1. It is well-known that antioxidants reduce harmful oxidative stress by reacting with free radicals and subsequently reduce inflammation [32,33]. These findings may be linked to the well-known antioxidant property of punicalagin [34]. Our results are promising and call for the development of further investigations to identify the molecular mechanisms behind the anti-atherosclerotic effects of punicalagin.

Peripheral circulating monocytes migrate into tissues during the inflammatory process as a critical pro-inflammatory response. In atherosclerosis, monocytes are related to hypercholesterolemia-associated migration to inflamed arteries, extravasation, sub-endothelial accumulation, and differentiation into macrophages [35]. MCP-1 is known to promote the migration and infiltration of monocytes/macrophages. In cardiovascular diseases, it has been demonstrated that MPC-1 deficiency results in a substantial reduction in arterial lipid deposition [36]. Our results presented on Figure 3 bring additional knowledge to this field of research, as we demonstrated that punicalagin considerably reduces MCP-1-induced monocyte migration. Taken together, these results could be essential for the prevention and the treatment of atherosclerosis.

On the other hand, anti-hyperlipidemic effect of punicalagin were examined in our study. Previous studies have mentioned the association of cholesterol efflux capacity in relation to atherosclerosis [37]. Accumulation of cholesterol in foam cell macrophages will result in plaque formation that leads to atherosclerosis [38,39,40]. Our data obtained from cholesterol efflux assay has demonstrated the capacity of punicalagin to enhance cholesterol efflux in IFN-γ-induced THP-1 macrophages. This will probably give a clue on how punicalagin acts as an anti-atherosclerotic agent.

## 4. Materials and Methods 

### 4.1. Reagents

For all experiments, Roswell Park Memorial Institute (RPMI) 1640 culture medium Cat No. A1049101; Fetal bovine serum Cat No. A3160802; Penicillin-Streptomycin Cat No. 15140122; 2-mercaptoethanol Cat No. M6250-10ML, l-Glutamine (200 mM) Cat No. 25030081, Phosphate buffered saline (PBS) solution Cat No. 10010015 and MCP-1 (MCAF) Recombinant Human Protein Cat No. PHC1014 were obtained from (Gibco-BRL, Cheshire, UK). Punicalagin (≥98% HPLC) Cat No. P0023, phorbol myristate acetate (PMA), human interferon-gamma and crystal violet were obtained from (Sigma-Aldrich, Gillingham UK). Dimethyl sulfoxide (DMSO) was obtained from (Invitrogen, Carlsbad, CA, USA). Pierce LDH Cytotoxicity Assay Kit was obtained from (Thermo Fisher Scientific, Waltham, MA, USA). RNeasy Mini Kit Cat No: 74104 and QuantiFast SYBR Green PCR Kit Cat No.: 204054 were obtained from (Qiagen, Manchester, UK). Im Prom-II Reverse Transcription System Cat No: A3800 was obtained from (Promega, Madison, WI, USA). 

### 4.2. Preparation of Punicalagin Treatment

Ten mg of punicalagin were diluted in 921 μL of DMSO to achieve a concentration of 10 mM. This main stock can be stored in the dark at −20 °C for at least six months.

### 4.3. THP-1 Cell Culture

THP-1 cells (monocytic leukemia cell lines) were obtained from the Molecular Biomedicine Unit at King Faisal Specialist Hospital and Research Centre (KFSHRC) in Riyadh, Saudi Arabia. Upon receiving the cells, they were sub-cultured in suspension in T75 and/or T25 flasks in a vertical position. RPMI-1640 was supplemented with antibiotics (100 μg/mL of both streptomycin and penicillin) and 10% fetal bovine serum (FBS). THP-1 cells were sub-cultured every 48–72 h. Trypan blue assay was used to examine cell viability.

### 4.4. Lactate Dehydrogenase (LDH) Assay

THP-1 monocytes were seeded in 96-well plates at densities of 105 cells/cm^2^ and differentiated by supplementing the RPMI medium with 160 nM of PMA. Differentiation of the seeded cells into related macrophages occurred overnight in an incubator at a humidity of 5% carbon dioxide *v*/*v*. To initiate foam cell formation, 130 nM of IFN-γ were added for 3 h to one set of the samples and vehicle in the other. Subsequently, the differentiated cells were exposed to 10 μM of punicalagin for another 24 h. All cells from the positive control well were lysed using the lysis buffer from the LDH kit for 45 min. A negative control was used for background reading. Using a 96-well plate, 50 μL volume of the well’s content solution was removed into a new 96 well plate and mixed using 50 μL of assay buffer. After 30 min of incubation at room temperature, 50 μL of stopping solution was added. Absorption was read at 570 nm using a BioTek plate reader (BioTek Instruments, Winooski, VT, USA). The negative values of the control were deducted from actual readings and read as percentage of cell viability in comparison to the experimental control.

### 4.5. Crystal Violet Assay

For the crystal violet assay, we used macrophages from the LDH assay to meet the experimental conditions. Remaining culture media were discarded from the original 96 well plate obtained from LDH assay and 50 μL of 0.2% (*w*/*v*) crystal violet solution (containing 10% ethanol) were added for 5 min at room temperature to stain the cells. Macrophages were washed at least three times in PBS before adding 50 μL of a solubilization buffer (containing 0.1 M NaH_2_PO_4_ dissolved in ethanol). The treated plate was aired on a shaking stand for 5 min before using a microplate reader at 570 nm to read its absorbance. The crystal violet bound DNA of adherent cells, that are considered as live cells. Results are expressed as the percentage of viability compared to control conditions.

### 4.6. RNA Extraction

For RNA extraction, we initiated differentiation from monocytes to macrophages by seeding THP-1 monocytes in six T25 flasks and treating all cells with PMA. The differentiation process using PMA occurred over 24 h in an incubator, at a humidity of 5% carbon dioxide *v*/*v*. Following incubation, one flask was treated with DMSO only as a control and second one was treated with IFN-γ only for 3 h. The four other flasks were treated with 5 or 10 µM of punicalagin in the absence or presence of IFN-γ. Twenty hours later, cells were harvested and RNeasy kit (Qiagen, Valencia, CA, USA) was used to extract RNA according to the manufacturer specifications. RNA concentration was measured using a NanoDrop ND 1000 spectrophotometer (ThermoFisher Scientific, Wilmington, DE, USA), while RNA purity was specified using the A260/A280 ratio. A ratio between 1.8 and 2.1 indicated high-purity RNA.

### 4.7. Analysis of Gene Expression Using qRT-PCR

Complementary DNA (cDNA) was prepared via reverse transcription of mRNA using the ImProm-II Reverse Transcription kit (Promega, Madison, WI, USA), according to the manufacturer’s protocol. For long-term storage, cDNA products were kept frozen at −80 or −20 °C. Quantitative PCR was performed using cDNA in a PCR 96-well plate from (Applied Biosystems, Beverly, MA, USA) and the SYBR Green Kit (Qiagen, Manchester, UK) according to the manufacturer’s protocol. StepOnePlus real-time-polymerase chain reaction (RT-PCR) System (Applied Biosystems, Waltham, MA, USA) was used to run the PCR.

The genes of interest were MCP-1 and ICAM-1. Glyceraldehyde-3-phosphate dehydrogenase (GAPDH) was used as a reference gene. Primer sequences of genes are as follows in (Table 1): 

PCR reactions were completed using the following steps: denaturation occurred at 94 °C for 120 s, and 40 amplification cycles of denaturation (30 s at 95 °C)—annealing step (60 s at 60 °C)—extension step (60 s at 72 °C) [41]. Messenger RNA expression changes of MCP-1 and ICAM-1 were compared to the internal housekeeping control GAPDH and calculated using the Ct relative gene expression method [42]. The values were analyzed using Microsoft Excel 2010 and GraphPad Prism version 8 software (San Diego, CA, USA).

### 4.8. MCP-1-Induced Monocyte Migration Assay

Monocyte migration in response to MCP-1 was investigated using a Boyden chamber assay as previously described [43]. An insert containing a membrane with 8 μm pores was used to split wells into separate halves to mimic the arterial endothelium layer, while allowing monocytes migration. To induce migration, 20 ng/mL of MCP-1 chemokine or DMSO was added to a total of 1 mL culture media to the bottom half of all the wells. Undifferentiated THP-1 monocytes (5 × 105 cells/well) were added to the top half of the well. Culture media, either containing punicalagin or DMSO, was then added to the top halves of the wells until a total volume of 0.5 mL was achieved. The modified Boyden chambers were then incubated at 37 °C in 5% (*v*/*v*) CO2 for 3 h. 

The media in the top half of the wells was then removed, and the underside of the membrane washed with 0.5 mL PBS to remove any adherent cells. The media in the bottom of the wells was then transferred to a 10 mL Falcon tube and centrifuged at 250× *g* for 5 min at room temperature. Cells were resuspended in 2 mL of culture media and counted using a hemocytometer. The number of cells that had migrated from the top to the bottom half of the well was calculated and expressed as a percentage of the number of cells that had been originally added.

### 4.9. Cholesterol Efflux Assay

To examine the effect of punicalagin on the progression of atherosclerosis in our cellular model, cholesterol efflux was performed using commercial Cholesterol Efflux Assay Kit Cat No.: ab196985 from (Abcam, UK). THP-1 monocytes were counted and 1 × 10^5^ cells/well were seeded in a 96-well black plate with clear bottom using 100 μL media/well and incubated for one hour to settle down. Then, 160 nM of PMA were added to allow differentiation into adherent macrophages and cells were incubated overnight. To initiate foam cell formation, 130 nM of IFN-γ was added for 3 h to one set of the samples and vehicle in the other. After incubation, cell monolayer was washed with serum free RPMI 1640 media. Labeling Reagent and Equilibration Buffer (provided in the kit) were mixed just before use. One hundred microliters per well were added to cells. After overnight incubation, Labeling Reagent was removed, and cells were washed gently by adding 200 μL of serum free RPMI. One hundred microliters of fresh RPMI containing 10 μM punicalagin were added to each well in both sets of samples. Cells were incubated for 24 h in a 37 °C incubator containing 5% CO_2_. 

For wells of positive control, 20 μL of Positive Control (provided in the kit) were added to 80 μL of RPMI while 100 μL of serum free RPMI were added to negative control wells. Cells were incubated for another 4 h in a 37 °C incubator containing 5% CO_2_. Then, supernatant was transferred to a new 96-well plate while cell monolayer was solubilized by 100 μL of cell lysis buffer and put on a plate shaker for 30 min at room temperature. Fluorescence of the two resulting plates were measured using Ex/Em = 482/515 nm. The percentage of cholesterol efflux was calculated by dividing the fluorescence intensity of the medium by the total of fluorescence intensity of medium and cell lysate.

(1) $$\%\;\mathrm{Cholesterol}\;\mathrm{Efflux}=\frac{\mathrm{Fluorescence}\;\mathrm{Intensity}\;\mathrm{of}\;\mathrm{Media}}{\mathrm{Fluorescence}\;\mathrm{Intensity}\;\mathrm{of}\;\mathrm{Media}+\mathrm{Cell}\;\mathrm{Lysate}}\;\times \;100$$

### 4.10. Statistical Analysis

Statistical analysis was performed using a one-way ANOVA followed by a Sidak post-hoc analysis. Software used for statistical analysis were Excel Microsoft 2010 and GraphPad Prism version 8.

## 5. Conclusions

The results obtained during the present study indicate that punicalagin regulates several key processes related to atherosclerosis. These findings are demonstrated by punicalagin’s inhibition of IFN-γ-induced MCP-1 and ICAM-1 gene expression at mRNA level and reduction of MCP-1 mediated monocyte migration and enhancement of cholesterol efflux from macrophages in vitro. Our current results suggest that punicalagin could be of interest to prevent atherosclerosis development and further in vivo research would be important.

## Figures and Tables

**Table 1:** Primer Sequences of Genes Used in RT-qPCR.

Genes	Forward Sequence (5′-3′)	Reverse Sequence (5′-3′)
H-GAPDH	CTTTTGCGTCGCCAGCCGAG	GCCCAATACGACCAAATCCGTTGACT
H-MCP-1	CGCTCAGCCAGATGCAATCAATG	ATGGTCTTGAAGATCACAGCTTCTTTGG
H-ICAM-1	GACCAGAGGTTGAACCCCAC	GCGCCGGAAAGCTGTAGAT
